# Osteomyelitis Risk in Patients With Transfemoral Amputations Treated With Osseointegration Prostheses

**DOI:** 10.1007/s11999-017-5507-2

**Published:** 2017-09-22

**Authors:** Jonatan Tillander, Kerstin Hagberg, Örjan Berlin, Lars Hagberg, Rickard Brånemark

**Affiliations:** 1 Department of Infectious Diseases, Institution of Biomedicine, University of Gothenburg, Gothenburg, 416 45 Sweden; 2 Department of Orthopaedics, University of Gothenburg, Gothenburg, Sweden; 3 Department of Orthopaedic Surgery, University of California, San Francisco, CA USA; 4 Advanced Reconstruction of Extremities, Sahlgrenska University Hospital, Gothenburg, Sweden

## Abstract

**Background:**

Percutaneous anchoring of femoral amputation prostheses using osseointegrating titanium implants has been in use for more than 25 years. The method offers considerable advantages in daily life compared with conventional socket prostheses, however long-term success might be jeopardized by implant-associated infection, especially osteomyelitis, but the long-term risk of this complication is unknown.

**Questions/Purposes:**

(1) To quantify the risk of osteomyelitis, (2) to characterize the clinical effect of osteomyelitis (including risk of implant extraction and impairments to function), and (3) to determine whether common patient factors (age, sex, body weight, diabetes, and implant component replacements) are associated with osteomyelitis in patients with transfemoral amputations treated with osseointegrated titanium implants.

**Methods:**

We retrospectively analyzed our first 96 patients receiving femoral implants (102 implants; mean implant time, 95 months) treated at our center between 1990 and 2010 for osteomyelitis. Six patients were lost to followup. The reason for amputation was tumor, trauma, or ischemia in 97 limbs and infection in five. All patients were referred from other orthopaedic centers owing to difficulty with use or to be fitted with socket prostheses. If found ineligible for this implant procedure no other treatment was offered at our center. Osteomyelitis was diagnosed by medical chart review of clinical signs, tissue culture results, and plain radiographic findings. Proportion of daily prosthetic use when osteomyelitis was diagnosed was semiquantitatively graded as 1 to 3. Survivorship free from implant- associated osteomyelitis and extraction attributable to osteomyelitis respectively was calculated using the Kaplan-Meier estimator. Indication for extraction was infection not responsive to conservative treatment with or without minor débridement or loosening of implant.

**Results:**

Implant-associated osteomyelitis was diagnosed in 16 patients corresponding to a 10-year cumulative risk of 20% (95% CI 0.12–0.33). Ten implants were extracted owing to osteomyelitis, with a 10-year cumulative risk of 9% (95% CI 0.04–0.20). Prosthetic use was temporarily impaired in four of the six patients with infection who did not undergo implant extraction. With the numbers available, we did not identify any association between age, BMI, or diabetes with osteomyelitis; however, this study was underpowered on this endpoint.

**Conclusion:**

The increased risk of infection with time calls for numerous measures. First, patients should be made aware of the long-term risks, and the surgical team should have a heightened suspicion in patients with method-specific presentation of possible infection. Second, several research questions have been raised. Will the surgical procedure, rehabilitation, and general care standardization since the start of the program result in lower infection rates? Will improved diagnostics and early treatment resolve infection and prevent subsequent extraction? Although not supported in this study, it is important to know if most infections arise as continuous bacterial invasion from the skin and implant interface and if so, how this can be prevented?

**Level of Evidence:**

Level IV, therapeutic study.

## Introduction

**Fig. 1:**
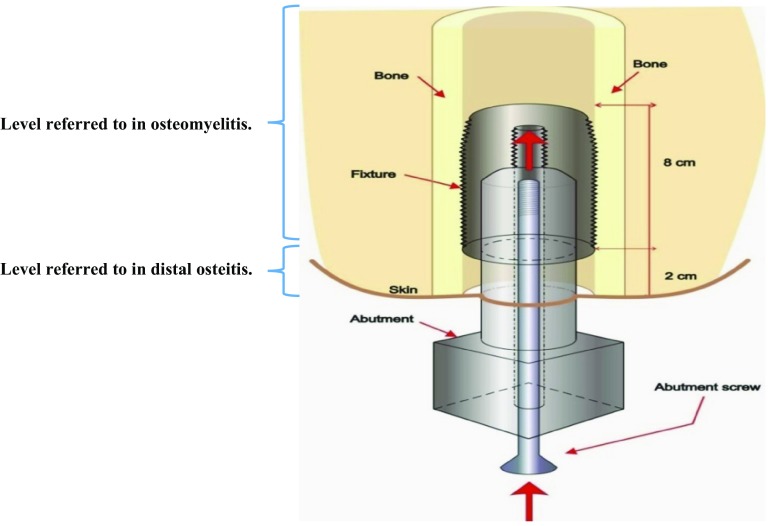
The schematic shows the implant components and tissues of the femoral residual limb. The level of implant-associated osteomyelitis and distal osteitis respectively, is indicated by brackets. (Published with permission from Cecilia Berlin PhD, Chalmers University of Technology, Gothenburg, Sweden. Illustration licensed under Creative Commons BY 4.0.).

Patients with transfemoral amputations traditionally have been treated with a prosthesis incorporating a socket in which the residual limb is placed, along with some form of supportive girdle to permit motion and secure fit. For many patients socket use is complicated by loading pain or sitting discomfort, contact dermatitis or sores, and reduced ROM [15]. In addition, patients with short stumps may have difficulty with fit and function. As a result of these issues, Brånemark et al. [9] expanded the application of osseointegrated dental implants [7] to limb replacement for patients unable to be fitted with, or use a socket. Osseointegration is recognized as the approximation of the implant titanium oxide surface and bone tissue with no interposition of fibrous tissues [12]. Briefly, the surgical method involves a threaded titanium cylinder (fixture) inserted in the medullary cavity of the residual bone. Undisturbed integration is allowed for 6 months before a skin-penetrating extension (abutment) is inserted in the fixture (Fig. 1), serving as an anchor site for the external prosthesis. This is followed by a carefully designed rehabilitation program [16]. There is no skin attachment to the abutment, thus it can be replaced if bent or damaged. In selected patients with amputation (mainly attributable to trauma and tumor), the method results in improved prosthesis handling and limb control, eliminates socket-caused skin disorders, and improves quality of life [17, 26, 42]. Overall cost effectiveness compared with socket-suspended prostheses is not known, but annual mean prosthetic workshop costs are similar [18]. The principle is well established in the prosthetic replacement of teeth [7] and in craniofacial reconstruction [14]. In lower limb replacement a few modular methods [9, 23, 43] were developed after the first orthopaedic implantation by Per Ingvar Brånemark and Björn Rydevik in 1990 [9]. The use of biomedical implants involves a risk of infection, particularly if the skin barrier is penetrated. Bacterial adhesion with subsequent biofilm formation are considered central in foreign-body infection and in the compromised tissues of other chronic infections [11]. Most bacteria have these properties, but coagulase-negative staphylococci and *Staphylococcus aureus* dominate in human implant infections [1, 24]. Other important biofilm-producing bacteria include *Enterococcus faecalis* and *Propionibacterium acne* [20, 30]. Risk factors for prosthetic joint infections include diabetes, rheumatoid arthritis, renal failure, malnutrition, immunosuppression, wound infection, and the nasal carriage of *S aureus* [5]. Obesity has been shown to be an independent risk factor for periprosthetic infection [32]. Smoking is detrimental to bone healing and diabetes appears to impede osseointegration [4, 19, 29, 37]. At our center, preoperative selection includes ruling out patients with suspected infection, and diagnostic biopsies have been introduced to this end. Bacteria may enter deep tissues at implantation, through the skin breach, the central screw canal of the fixture, or by the hematogenous route, theoretically making this method vulnerable to superficial and deep infection. However, the proximal end of the fixture is sealed by the air-tight central screw (which can be removed for marrow blood sampling) and adequate osseointegration counteracts bacterial spread along the outer threads of the fixture [9, 10]. Titanium interacts more favorably with surrounding bone than conventionally used implant metals. This appears to reduce the risk of bone infection [13, 38].

Overall outcome for the first 100 patients with a transfemoral amputation indicated a higher success rate with newer treatment protocols and greater surgical experience, but failures relating to infections were not systematically evaluated [16]. Branemark et al. [8] reported that four of 51 patients experienced a deep infection during a 2-year period. Previous prospective data from our center in a similar but smaller cohort suggested that 18% of patients have an implant-associated deep infection during a 3-year period [40]. Many of these infections showed little inflammatory activity and involved only limited and temporary loss of function for the patient [40]. *S aureus* was slightly more common than coagulase-negative staphylococci followed by *Enterococcus* spp. in diagnostic cultures.

This method, intended for life-long prosthetic limb support, is potentially vulnerable to infection limiting its usefulness. Therefore, we aimed to (1) quantify the risk of osteomyelitis, (2) characterize the clinical effect of osteomyelitis (including the risk of prosthesis extraction and impairments to function), and (3) determine whether common patient factors (age, sex, body weight, diabetes, and implant component replacements) are associated with osteomyelitis in patients with transfemoral amputations treated with osseointegrated titanium implants.

## Patients and Methods

We performed a retrospective cohort study of the first 96 patients with transfemoral amputations selected for treatment with 102 (six bilateral) intramedullary transcutaneous titanium implants at our osseointegration center in Gothenburg between May 1990 and January 2010 (Table 1). All patients were referred from other Swedish or European centers owing to difficulty to use (socket complications) or be fitted with (stump malformation) a socket prosthesis. No alternative treatment was offered at our center for the approximately one-third of the patients who were not found suitable for implant surgery in team evaluation. Twenty-seven patients had their implants before the start of a systematic treatment and rehabilitation protocol (Osseointegrated Prostheses for the Rehabilitation of Amputees [OPRA]), yielding prospective, nonrandomized results on overall outcome and patient-centered function [8] in January 1999. Fifty-one patients were treated in the OPRA study, and 18 were treated after that, without any protocol alterations. Ninety-one patients underwent amputation owing to tumor, trauma, or an ischemic event, and five underwent amputation because of primary deep-seated infection. Apart from eight patients (six with diabetes and peripheral artery disease, one with severe neurofibromatosis, one with cytostatic treatment), all patients were free from other major illnesses at the time of implant surgery. Limbs were grouped according to a previously suggested standard of residual lengths [34].

**Table 1:** Basic demographics at implant surgery

Demographic	Value
Number of patients (men/women)	96 (60/36)
Number of implants (bilateral implants)	102 (6)
Reasons for amputation: tumor/trauma/ischemia/infection/other	20/71/5/5/1
Time since amputation, mean (range)	11.5 (< 1–44) years
Age, mean (range)	43.5 (19–65) years
BMI, mean (range)*	26 (16-43) kg/m^2^
Number of smokers	22
Number of patients with diabetes (insulin dependent)	6 (3)
Residual limb lengths, short/normal/long	34/60/8

*Thirteen patients had Class I obesity (BMI 30–34.9 kg/m^2^) and four had obesity Classes 2 and 3 (BMI, 35 to ≥ 40 kg/m^2^).

**Fig. 2A–B:**
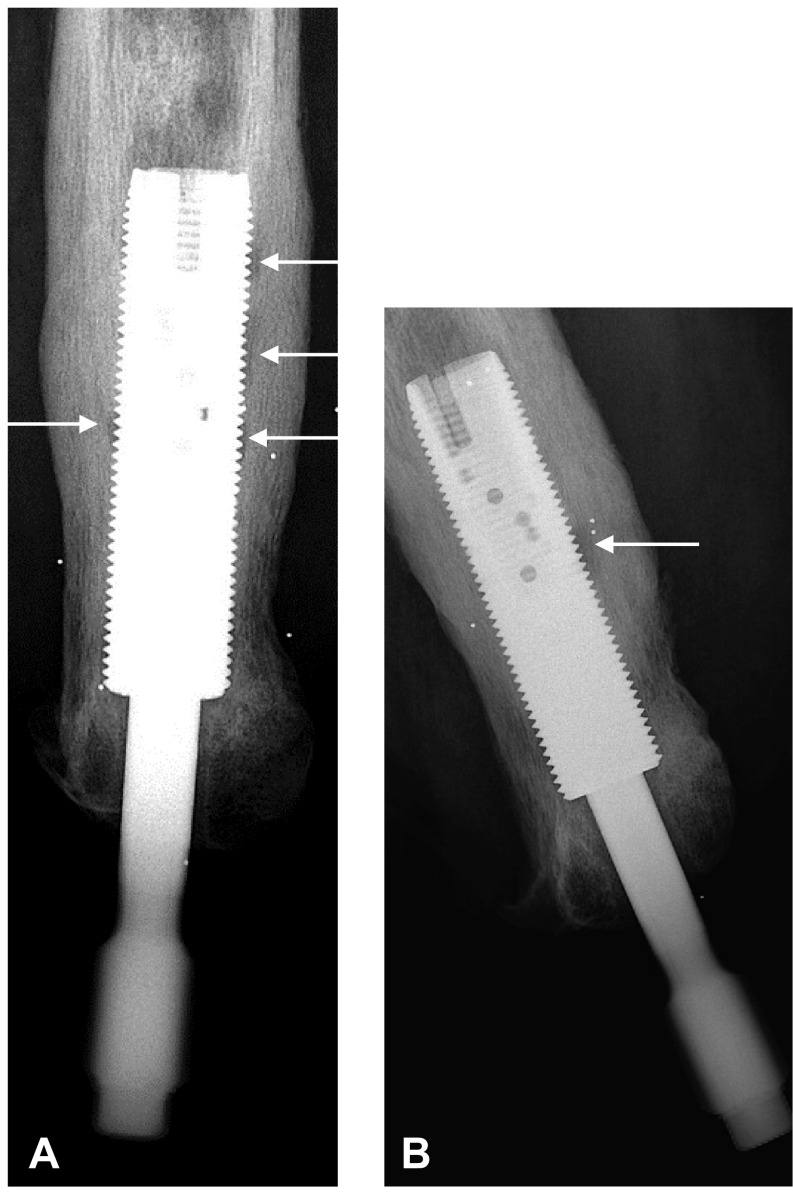
**(A)** AP and **(B)** lateral view plain radiographs show small zones of radiolucency (arrows) between the implant and bone in a male patient with osteomyelitis around an osseointegrated implant in the left femur. Free projection of the implant threads is important for correct evaluation.

The time from implant insertion to the diagnosis of osteomyelitis and/or extraction caused by infection was registered. The mean observation time in the study was 7.9 years (median, 6.2 years; range, 1.5–19.6 years). Diagnoses were based on careful examinations of patient records, culture results [3], and plain radiographs (Fig. 2) examinations. Osteomyelitis was defined as evidence of infection involving implant-surrounding bone and/or bone marrow, supported by positive percutaneous bone biopsy or aspirated bone marrow cultures, and classified as definite, probable, or possible (Table 2). The definitions are based on diagnostic algorithms for prosthetic joint infections [3, 39]. Infections were considered resolved if patients were symptom-free 12 months or more after discontinuation of antibiotics. Bone marrow is accessible via the normally screw-sealed proximal end of the intramedullary component. Peripheral blood cultures were not routinely taken. Indication for extraction was infection not responsive to conservative treatment or loosening evident in stability testing of the implant. Eight patients were right-censored in the implant survival analyses for reasons other than study completion (five for noninfected implant extractions [including reimplantations]; one lost to followup; one with a retained fixture and sealed skin; and one death not related to the implant). Local signs of infection and bone attrition rather than osteolysis in the femoral shaft distal to the fixture were difficult to define. Microbial involvement was highly suspected, but an etiologic diagnosis (ie, bacterial osteitis) was unattainable owing to lack of reliable tissue cultures. This distal osteitis was separately registered. Prosthetic use at the time of osteomyelitis was retrospectively assessed by a team physiotherapist (KH) and assigned a simple 1 to 3 score (unchanged = 1, impaired = 2, and no prosthetic use owing to infection = 3). Patients still in the early postoperative rehabilitation phase (the first months before use of the prosthesis in daily life) at the time of the infection were not assessed in terms of impaired prosthetic use.

**Table 2:** Definitions of osteomyelitis around the implant system

Type of infection	Signs and symptoms*	Positive tissue cultures	Positive radiograph^#^
Definite implant infection	Yes	Yes^†^	Yes
Probable implant infection	Yes	Yes^‡^	Yes
Possible implant infection	Yes	No	Yes

*Loaded/unloaded pain, stump swelling, and purulent secretion with or without visible skin inflammation in the skin penetration area; ^†^intraoperative cultures where ≥ 2 of 5 yielded identical bacteria; ^‡^intraoperative cultures not meeting ^†^criteria; ^#^radiographic evidence of osteolysis with or without periosteal sclerosis around a previously integrated implant. In acute infection, negative findings were disregarded.

Furthermore, the total number of short courses of oral antibiotics for superficial infection in each patient was recorded. There was concern that pressure-induced bone marrow contamination could occur during the exchange of abutments and all exchanges therefore were recorded. Previously identified risk factors for impaired bone-implant healing and infection common in this cohort (ie, smoking, diabetes, [high] BMI, and age), were compiled from chart review and analyzed for association with osteomyelitis [4, 5, 19, 29, 32, 37].

### Statistics

Statistical end-points were first implant osteomyelitis and first implant extraction owing to infection. We used the Kaplan-Meier estimator to calculate the risk of osteomyelitis and extraction with time. Based on data at the time of implant insertion, risk factor correlation was performed with the Cox proportional hazard model. A hazard ratio (HR) for cumulative abutment replacements was obtained by time-modified Cox analysis. Differences were considered significant at a probability less than 0.05. Data were computed with the GraphPad Prism 6 (GraphPad Software Inc, San Diego, CA, USA) and SAS (SAS Institute Inc, Cary, NC, USA) statistical software.

## Results

### Long-term Risk of Osteomyelitis

**Fig. 3:**
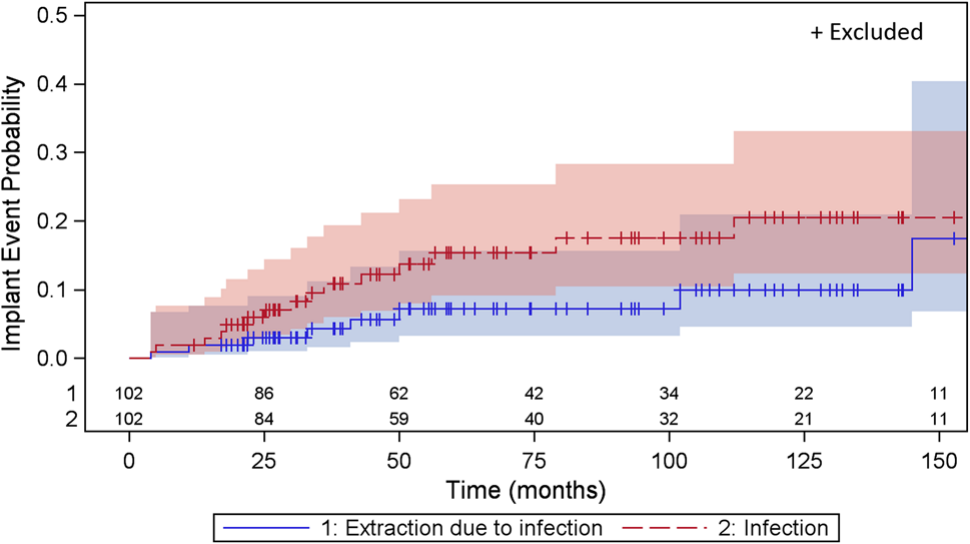
The graph shows the probability, with 95% CIs, of osteomyelitis and extraction owing to osteomyelitis with time.

By 10 years the estimated risk of osteomyelitis reached 20% (95% CI, 0.12–0.33) (Fig. 3). After that, the number of patients at risk was too low, making further estimation uncertain. The median time from implantation to osteomyelitis was 2.6 years (range, 0.3–13.8 years). During the entire 20-year study period, 16 patients (16 implants) had osteomyelitis (12 definitive, three probable, one possible) develop, which in 10 instances led to extraction of the implant translating to a 10-year cumulative risk of 9% (95% CI, 0.04–0.20). Seven of these patients were treated before the OPRA protocol. Osteomyelitis developed in five of 34 short stumps (15%), while figures for normal and long stumps were 10 of 60 (17%) and one of eight (13%). Six patients (one with bilateral implants) without definable osteomyelitis had signs of distal osteitis, as defined above, corresponding to an 8% (95% CI, 0.02–0.24) risk at 10 years of implant use (Fig. 2). No distal osteitis was clinically recognized before the 5-year followup after implant insertion (mean, 125.5 months; range, 64–192 months) and in the patient with bilateral implants, as late as 14 and 16 years respectively. Microbial involvement was highly suspected, but in the absence of reliable tissue cultures and histologic analyses, an etiologic diagnosis (ie, bacterial osteitis) was unattainable.

### Clinical Effect of Osteomyelitis

Of the 16 patients with a diagnosis of osteomyelitis, 10 required removal of the implant for complete recovery (Table 3). The clinical presentation of osteomyelitis varied, being either subacute or acute (eight patients), including one patient with mild septicemia, or chronic with or without fistulas (eight patients). There was a wide range in prosthetic use at the time of diagnosis of osteomyelitis. Two patients (later implant extraction in one) were unable to use their prostheses, six had moderately restricted prosthetic use (three implants extracted), and two had no impairment (no implant extracted). The remaining six patients were in the early rehabilitation phase and therefore could not be assessed. In this group, all six implants were extracted between 4 to 102 months (median, 25 months) from fixture insertion. In four patients, osteomyelitis was resolved with antibiotics without extracting the implant. Three of these patients had a staphylococcal infection and received a rifampin-based treatment. *S aureus* and coagulase-negative staphylococci were the most common bacteria isolated in intraoperatively obtained cultures (Table 4). In one patient, *S aureus* was isolated from peripheral blood after an episode of fever but no signs of severe sepsis such as manifest hypotension or organ dysfunction developed. In patients with distal osteitis the clinical course was milder, and none of the implants were extracted in this group. In five of six patients (seven implants) considered to have distal osteitis, skin infection was present at diagnosis and all had various other skin problems (ie, fibrous hyperplasia, hyperkeratosis, and ulcers). Three had unrestricted and three had moderately impaired prosthetic use at the time of diagnosis. The mean use of short-course antibiotics in patients with distal osteitis (mean number of courses, 21.5; range, 10–30) was more frequent (p < 0.01) compared with that of patients later diagnosed with implant infection (mean number of courses, 6; range, 0–15).

**Table 3:** Clinical outcome for patients classified as having osteomyelitis during the study period

Recovery after antibiotics with or without minor débridement (number of patients)	Recovery and later relapse (number of patients)	Successful reimplantation (number of patients)	Recovery after extraction (number of patients)	Chronic with fistula (number of patients)
4	1	1	9	1

**Table 4:** Bacterial yield by intraoperative bone or marrow cultures in patients with osteomyelitis

Bacteria	Number of isolates*
*Staphylococcus aureus*, including one case of methicillin-resistant *S aureus*	9
Coagulase-negative staphylococci, including one case of *Staphylococcus lugdunensis*	4
*Enterococcus faecalis*	2
*Escherichia coli*	1
*Proteus mirabilis*	1
*Peptostreptococci*	1
*Pseudomonas aeruginosa*	1
Negative	1

*In four infections, two bacterial species were isolated and in one, reliable cultures were lacking.

### Patient Factors Examined for Association With Osteomyelitis

With the numbers available, we found no association between selected patient factors and osteomyelitis. An increase in replacement of abutments was not related to implant infection (HR, 1.13; p = 0.16), and no risk increase was seen in patients who were overweight (BMI > 25 kg/m^2^) (HR, 0.99; 95% CI, 0.90–1.09; p = 0.97), elderly (HR, 0.98; 95% CI, 0.96–1.02; p = 0.75), or who smoked (HR, 1.8; 95% CI, 0.69–4.73; p = 0.22) during the time of implant surgery. Furthermore, there was no sex difference (HR, 0.92; 95% CI, 0.39–2.17; p = 0.85) or risk increase in patients with uncomplicated diabetes at the time of implant insertion (HR, 2.70; 95% CI, 0.78–9.39; p = 0.11).

## Discussion

In individuals with transfemoral amputations, especially with a short or malformed residual limb, socket prostheses cause substantial disadvantages for many including discomfort, skin problems, and poor function. These problems are reduced substantially when the prosthetic limb is attached to a percutaneous osseointegrated implant. However, the long-term risk of implant-associated infection and its clinical effect are not known with this method. In the first 96 patients treated with this novel method, we estimated that the cumulative risk of implant-associated osteomyelitis was 20% and the risk of implant extraction attributable to infection was 9% during a 10-year implant period. In four of the six patients for whom infection did not lead to extraction, prosthetic use was impaired. Resolution of osteomyelitis was attainable with prolonged combined antibiotics in one-quarter of the patients.

### Limitations

Our study has several limitations. Comorbidities associated with risk of poor healing and infection (arterial disease and complicated diabetes) are underrepresented in the patient cohort owing to preoperative selection. BMI, uncomplicated diabetes, and smoking were recorded only at the time of implantation, and no adjustments were made owing to changes in these variables with time which would only permit detection of a very strong relationship. The data were extracted retrospectively from medical charts by one author (JT) and reexamined by all authors before being added to the final dataset. We acknowledge the difficulty of defining method-typical osteomyelitis. Our definitions, although not validated, avoid excluding patients with culture-negative results with a high overall likelihood of osteomyelitis. Any overestimation is likely small as osteomyelitis was definite in 75% of the patients. Furthermore, the reliability of the method-specific tissue sampling is not known. Although less invasive than percutaneous bone tissue biopsies, bone marrow aspirations through the fixture possibly are more vulnerable to sample contamination, and it is uncertain if multiple samples add to diagnostic precision, as contaminants and true pathogens alike disperse in the liquid phase. Functional impairment could be graded only approximately owing to the 20-year retrospective span. Six patients were lost to followup including one patient with method-unrelated death, but all patients from the early phase (1990–1998) of the method development (custom-design period) have been included. Since then, several modifications have been made leading to standardized implants, surgical technique, and a strict rehabilitation protocol. This may result in reduced infectious complications. Outcome improvement also can be expected with standardized diagnostic protocols and early treatment with biofilm-effective antibiotics [6, 33, 36].

This method, intended for life-long prosthetic limb support, is potentially vulnerable to infection limiting its usefulness. However, we had no data with respect to very long-term risk of osteomyelitis and the clinical effects thereof. Previous outcome results not centering on infection indicate higher success rates with newer treatment protocols and greater surgical experience [8, 16]. In the current study, we found a high long-term risk of osteomyelitis in a young (mean age at first surgery, 43.5 years) patient group with good health compared with the average patient undergoing arthroplasty [41]. This raises concerns regarding increasing risk of infection attributable to aging and related morbidities. Lower infection rates are needed before the indication can be widened. Currently, this treatment is only offered when socket prostheses are not an option, preexisting conditions likely to increase risk for failure are ruled out, and patients are made fully aware of the elevated risk of infectious complications. The method has similarities to percutaneous bone fixation and joint arthroplasty. In the latter, infection rates have decreased dramatically since its introduction [25], with a 10-year cumulative incidence of approximately 1.5% to 2%, using standardized infection control measures [39]. Frequent but limited osteitis in external pin fixation [27] underscores how readily a percutaneous device promotes infection. Beyond this however, differences in surgical technique and tissue-material interaction do not allow for meaningful comparison. Some investigators aim for a microbial barrier by skin attachment to the percutaneous component [22, 35]. Conversely, our method aims to minimize skin mobility and secondary inflammation [9], in addition to allowing easy component replacements for the patient and surgeon. *S aureus* was twice as common as coagulase-negative staphylococci, likely because the skin stoma in this method favors the invasion-prone *S aureus* and not owing to a higher proportion of blood borne inoculation [39].

Full or moderate prosthetic use was maintained in more than half of the patients with infections not subjected to later implant extraction. Our interpretation is that load-bearing ossointegration can be preserved even in local osteomyelitis, justifying attempts of conservative treatment.

In a few patients, the infectious complications were acute, involving bone and marrow, indicating blood seeding from distant loci. That no patient had severe sepsis is important. However, a method resulting in frequent use of antibiotics contributes to bacterial resistance and increases adverse drug events. A strict policy in perioperative antibiotic prophylaxis and skin infection treatment will partly address this issue. Based on time of diagnosis, osteomyelitis develops later, and fewer implants are removed owing to early infection with this method compared with prosthetic joint infections [21, 28]. This likely stresses the importance of good primary osseointegration, translating to painless performance and possibly protection against early infectious development [8]. The robustness of the osseointegration and antiinfective properties of the titanium (oxide) surface [2, 13] might explain the lack of infectious problems for many patients despite the intimacy between the skin microflora and the foreign material. However, it is conceivable that diagnostic delay in part explains the above, especially during rehabilitation, when diffuse pain indirectly related to increased prosthetic loading appears difficult to distinguish from pain caused by osteomyelitis. From these observations, deep infection should be suspected in rehabilitation that is delayed because of pain, even in absence of other signs of infection. Furthermore, low-grade infection can be very difficult to distinguish from aseptic loosening, much like in arthroplasties [31]. Although not validated for our method, we suggest that diagnostic algorithms based on clinical signs, radiology, histopathology, and multiple tissue cultures [3, 33] be used to aid in decision making. Patients with distal osteitis were difficult to define as having implant-associated infection or not. This was partly because tissue culturing had been avoided owing to concern that it would interfere negatively with tissue integration, and partly because biomechanical bone wear was expected in this region. Frequent antibiotic use in this group however, suggests infectious bone degradation and supports appropriate culturing. It is not yet known whether distal osteitis progresses to osteomyelitis involving proximal parts of the implant system in the long term, but no such observation was made in the current study. Whether distal osteitis should be treated differently or at all therefore is not clear. Prospective investigations are warranted.

In our cohort (age range, 19–65 years), increased age did not appear to be a risk factor for infection, whereas time since implantation clearly was. We were concerned that exchanges of abutments after component failure might cause bacterial contamination, but we found no correlation between frequent abutment changes and infection. However, in four patients, there was a temporal relationship between abutment change and the start of infectious complications, which calls for prospective studies and strict aseptic conditions while performing this procedure which now have been implemented in our protocol. Only three patients had insulin-dependent diabetes, and none of them was severely obese. Therefore, it is not to be interpreted that diabetes is a negligible risk factor in this method. It might be expected that patients with a short residual limb are more prone to infection owing to the rich bacterial flora in the groin and a higher risk of implant instability and poor tissue integration, but our data do not support these assumptions.

## Conclusion

The bone-anchored prosthesis using the osseointegration technique substantially improves quality of life after a transfemoral amputation [17]. The key question is whether these advantages outweigh the risk of deep or recurring superficial implant-associated infection.

We showed that the overall risk of implant osteomyelitis in patients who receive percutaneous osseointegrated implants after transfemoral amputation increases with time. This is a major problem, as the method is intended to be a lifetime solution for prosthetic support. Infections which do not lead to implant removal only moderately reduce prosthetic function, and with more than 20 years’ experience with the method, we believe that improved daily living outweighs the risks and inconvenience of treatment for most patients [26] in this respect. As in all other medical interventions, it is important to inform patients regarding possible infectious complications and the risk of implant extraction if severe infectious complications occur. Will the surgical procedure, rehabilitation, and general care standardization since the start of the program result in lower infection rates? Prospective studies are warranted. Will improved diagnostics guiding early treatment and better handling of system components reduce infectious complications? Although not directly supported in this study, continuous bacterial invasion from the skin-implant interface as a cause for osteomyelitis cannot be ruled out. Further studies of method-specific diagnostics and treatment, bacterial properties, and characteristics of the skin-penetration area should be done to address these issues.

## References

[1] Arciola CR, Campoccia D, Speziale P, Montanaro L, Costerton JW (2012). Biofilm formation in Staphylococcus implant infections: a review of molecular mechanisms and implications for biofilm-resistant materials. Biomaterials..

[2] Arens S, Schlegel U, Printzen G, Ziegler WJ, Perren SM, Hansis M (1996). Influence of materials for fixation implants on local infection: an experimental study of steel versus titanium DCP in rabbits. J Bone Joint Surg Br..

[3] Atkins BL, Athanasou N, Deeks JJ, Crook DW, Simpson H, Peto TE, McLardy-Smith P, Berendt AR (1998). Prospective evaluation of criteria for microbiological diagnosis of prosthetic-joint infection at revision arthroplasty: the OSIRIS Collaborative Study Group. J Clin Microbiol..

[4] Baig MR, Rajan M (2007). Effects of smoking on the outcome of implant treatment: a literature review. Indian J Dent Res..

[5] Berbari EF, Hanssen AD, Duffy MC, Steckelberg JM, Ilstrup DM, Harmsen WS, Osmon DR (1998). Risk factors for prosthetic joint infection: case-control study. Clin Infect Dis..

[6] Betsch BY, Eggli S, Siebenrock KA, Tauber MG, Muhlemann K (2008). Treatment of joint prosthesis infection in accordance with current recommendations improves outcome. Clin Infect Dis..

[7] Branemark PI, Hansson BO, Adell R, Breine U, Lindstrom J, Hallen O, Ohman A (1977). Osseointegrated implants in the treatment of the edentulous jaw: experience from a 10-year period. Scand J Plast Reconstr Surg Suppl..

[8] Branemark R, Berlin O, Hagberg K, Bergh P, Gunterberg B, Rydevik B (2014). A novel osseointegrated percutaneous prosthetic system for the treatment of patients with transfemoral amputation: a prospective study of 51 patients. Bone Joint J..

[9] Branemark R, Branemark PI, Rydevik B, Myers RR (2001). Osseointegration in skeletal reconstruction and rehabilitation: a review. J Rehabil Res Dev..

[10] Branemark R, Thomsen P (1997). Biomechanical and morphological studies on osseointegration in immunological arthritis in rabbits. Scand J Plast Reconstr Surg Hand Surg..

[11] Donlan RM, Costerton JW (2002). Biofilms: survival mechanisms of clinically relevant microorganisms. Clin Microbiol Rev..

[12] *Dorland’s Illustrated Medical Dictionary.* 32nd ed. Philadelphia, PA: Elsevier; 2012.

[13] Gotman I (1997). Characteristics of metals used in implants. J Endourol..

[14] Granstrom G (2007). Craniofacial osseointegration. Oral Dis..

[15] Hagberg K, Branemark R (2001). Consequences of non-vascular trans-femoral amputation: a survey of quality of life, prosthetic use and problems. Prosthet Orthot Int..

[16] Hagberg K, Branemark R (2009). One hundred patients treated with osseointegrated transfemoral amputation prostheses: rehabilitation perspective. J Rehabil Res Dev..

[17] Hagberg K, Hansson E, Branemark R (2014). Outcome of percutaneous osseointegrated prostheses for patients with unilateral transfemoral amputation at two-year follow-up. Arch Phys Med Rehabil..

[18] Haggstrom EE, Hansson E, Hagberg K (2013). Comparison of prosthetic costs and service between osseointegrated and conventional suspended transfemoral prostheses. Prosthet Orthot Int..

[19] Hasegawa H, Ozawa S, Hashimoto K, Takeichi T, Ogawa T (2008). Type 2 diabetes impairs implant osseointegration capacity in rats. Int J Oral Maxillofac Implants..

[20] Holmberg A, Lood R, Morgelin M, Soderquist B, Holst E, Collin M, Christensson B, Rasmussen M (2009). Biofilm formation by Propionibacterium acnes is a characteristic of invasive isolates. Clin Microbiol Infect..

[21] Huotari K, Peltola M, Jamsen E (2015). The incidence of late prosthetic joint infections: a registry-based study of 112,708 primary hip and knee replacements. Acta Orthop..

[22] Jeyapalina S, Beck JP, Bachus KN, Williams DL, Bloebaum RD (2012). Efficacy of a porous-structured titanium subdermal barrier for preventing infection in percutaneous osseointegrated prostheses. J Orthop Res..

[23] Juhnke DL, Beck JP, Jeyapalina S, Aschoff HH (2015). Fifteen years of experience with Integral-Leg-Prosthesis: cohort study of artificial limb attachment system. J Rehabil Res Dev..

[24] Kloos WE, Bannerman TL (1994). Update on clinical significance of coagulase-negative staphylococci. Clin Microbiol Rev..

[25] Lidgren L (2001). Joint prosthetic infections: a success story. Acta Orthop Scand..

[26] Lundberg M, Hagberg K, Bullington J (2011). My prosthesis as a part of me: a qualitative analysis of living with an osseointegrated prosthetic limb. Prosthet Orthot Int..

[27] Mahan J, Seligson D, Henry SL, Hynes P, Dobbins J (1991). Factors in pin tract infections. Orthopedics..

[28] Malchau H, Herberts P, Eisler T, Garellick G, Soderman P (2002). The Swedish Total Hip Replacement Register. J Bone Joint Surg Am..

[29] Mellado-Valero A, Ferrer Garcia JC, Herrera Ballester A, Labaig Rueda C. Effects of diabetes on the osseointegration of dental implants. *Med Oral Patol Oral Cir Bucal.* 2007;12:E38–43. 17195826

[30] Mohamed JA, Huang DB (2007). Biofilm formation by enterococci. J Med Microbiol..

[31] Moojen DJ, van Hellemondt G, Vogely HC, Burger BJ, Walenkamp GH, Tulp NJ, Schreurs BW, de Meulemeester FR, Schot CS, van de Pol I, Fujishiro T, Schouls LM, Bauer TW, Dhert WJ (2010). Incidence of low-grade infection in aseptic loosening of total hip arthroplasty. Acta Orthop..

[32] Namba RS, Paxton L, Fithian DC, Stone ML (2005). Obesity and perioperative morbidity in total hip and total knee arthroplasty patients. J Arthroplasty..

[33] Osmon DR, Berbari EF, Berendt AR, Lew D, Zimmerli W, Steckelberg JM, Rao N, Hanssen A (2013). Wilson WR; Infectious Diseases Society of America. Diagnosis and management of prosthetic joint infection: clinical practice guidelines by the Infectious Diseases Society of America. Clin Infect Dis..

[34] Persson BM, Liedberg E (1983). A clinical standard of stump measurement and classification in lower limb amputees. Prosthet Orthot Int..

[35] Pitkin M, Raykhtsaum G, Galibin OV, Protasov MV, Chihovskaya JV, Belyaeva IG (2006). Skin and bone integrated prosthetic pylon: a pilot animal study. J Rehabil Res Dev..

[36] Puhto AP, Puhto T, Syrjala H (2012). Short-course antibiotics for prosthetic joint infections treated with prosthesis retention. Clin Microbiol Infect..

[37] Sloan A, Hussain I, Maqsood M, Eremin O, El-Sheemy M (2010). The effects of smoking on fracture healing. Surgeon..

[38] Suska F, Esposito M, Gretzer C, Kalltorp M, Tengvall P, Thomsen P (2003). IL-1alpha, IL-1beta and TNF-alpha secretion during in vivo/ex vivo cellular interactions with titanium and copper. Biomaterials..

[39] Tande AJ, Patel R (2014). Prosthetic joint infection. Clin Microbiol Rev..

[40] Tillander J, Hagberg K, Hagberg L, Branemark R (2010). Osseointegrated titanium implants for limb prostheses attachments: infectious complications. Clin Orthop Relat Res..

[41] Tsaras G, Osmon DR, Mabry T, Lahr B, St Sauveur J, Yawn B, Kurland R, Berbari EF (2012). Incidence, secular trends, and outcomes of prosthetic joint infection: a population-based study, Olmsted county, Minnesota, 1969-2007. Infect Control Hosp Epidemiol..

[42] Van de Meent H, Hopman MT, Frolke JP (2013). Walking ability and quality of life in subjects with transfemoral amputation: a comparison of osseointegration with socket prostheses. Arch Phys Med Rehabil..

[43] van Eck C, McGough RL (2015). Clinical outcome of osseointegrated prostheses for lower extremity amputations: a systematic review of the literature. Current Orthopaedic Practice..

